# Chemical tags and beyond: Live‐cell protein labeling technologies for modern optical imaging

**DOI:** 10.1002/smo.20230002

**Published:** 2023-08-28

**Authors:** Dilizhatai Saimi, Zhixing Chen

**Affiliations:** ^1^ College of Future Technology Institute of Molecular Medicine National Biomedical Imaging Center Beijing Key Laboratory of Cardiometabolic Molecular Medicine Peking University Beijing China; ^2^ Peking‐Tsinghua Center for Life Science Academy for Advanced Interdisciplinary Studies Peking University Beijing China

**Keywords:** fluorescent dyes, live‐cell imaging, protein labeling, self‐labeling tags, unnatural amino acids

## Abstract

Imaging proteins with high resolution is crucial for studying cellular physiology and pathology. Fluorescence imaging is a privileged method to visualize proteins with subcellular precision in live cells. In recent years, there has been a tremendous advance in the field of fluorescent dyes that are optically more sophisticated than genetically‐encodable fluorescent proteins. In this review, we aim to discuss modern bioconjugation methods to specifically incorporate these dyes into protein‐of‐interests. We focus on advances in live‐cell labeling strategies and fluorescent probes, especially the HaloTag, SNAP‐tag, TMP‐tag, and unnatural amino acid systems and their applications. These protein labeling methods, along with cutting‐edge dyes and novel microscopy methods, have become the infrastructure for biological research in the era of super‐resolution imaging.

## Graphical abstract

Rendering fluorescence to proteins is instrumental for modern bioimaging. This review summarizes the protein tags which can direct externally applied synthetic dyes to selected cellular proteins. Thanks to the superior optical properties of dyes compared to fluorescent proteins, protein tags are becoming a mainstay in the application of advanced fluorescent imaging methods to image live cells, tissues, and animals.

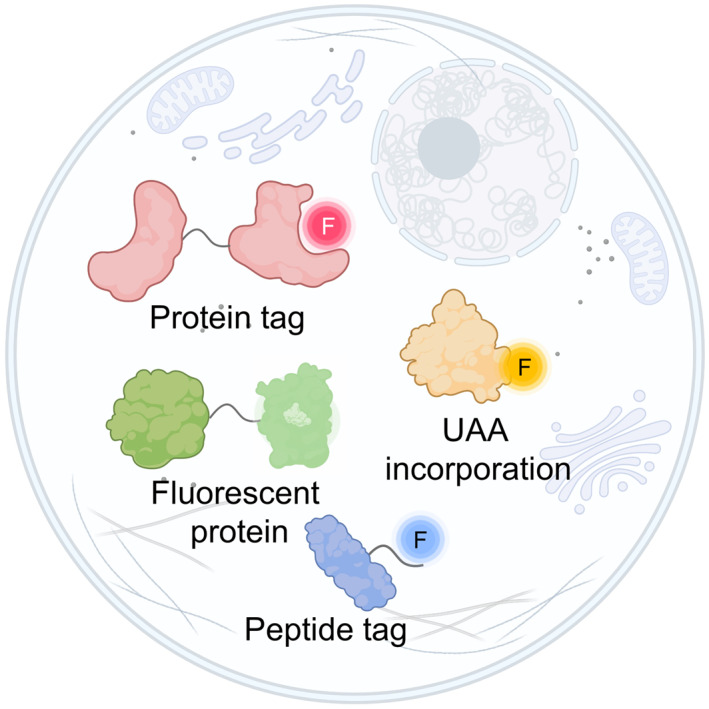

## 1 INTRODUCTION

Cells serve as the fundamental unit of analysis for a great deal of research in the field of life sciences. In the meantime, cells are a complex system with a series of biological macromolecules arranged orderly in space and time. Visualization of biomacromolecules such as proteins facilitates scientists to understand the interior work in cells and organisms. Among all the imaging systems currently available, light microscopy is particularly suitable for biological research. Fluorescence imaging, with its non‐invasive, multicolor and high‐resolution advantages, has enabled biological research to enter a brand‐new field of comprehending cellular processes at the single‐cell level.^[1]^ Labeling fluorescent probes onto proteins of interest is an important area of research for studying various protein functions, protein‐cellular component interactions, and protein mobility.^[2]^ Because of the complexity and responsiveness of the cellular environment, it is difficult to label proteins quickly, deliberately, and covalently in vivo.^[3]^

**FIGURE 1:**
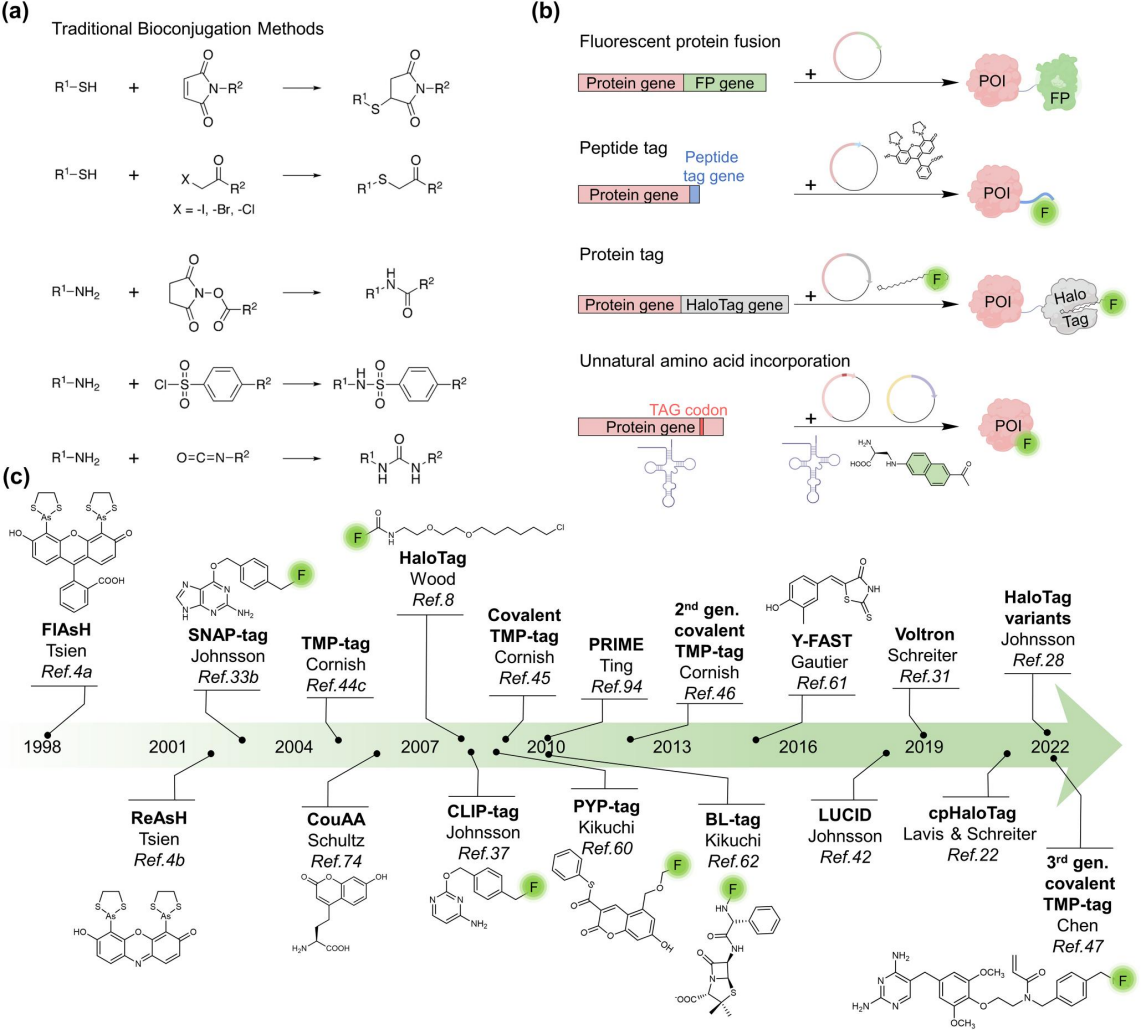
Methods for labeling proteins with fluorophores. (a) Traditional bioconjugation methods for the protein. (b) Labeling POI by various tools is presented. FPs, peptide tags, or HaloTag are genetically fused to a POI, and the HaloTag covalently binds to a fluorescent ligand. Fluorophore can be labeled to UAA. (c) Timeline of self‐labeling protein tags. Abbreviations: F, fluorophore; FP, fluorescent protein; POI, protein of interest; UAA, unnatural amino acid. The scheme in panel b was partially created with BioRender.com.

In traditional bioconjugation methods, the common targets among the different nucleophilic amino acid residues found in proteins are the thiol group of cysteine and the amino group of lysine (Figure 1a). Cysteine can be easily alkylated by reaction with suitable electrophilic reagents such as *a*‐haloketone or Michael receptors (e.g., maleimide derivatives). If the cysteine is not present on the surface of the protein, a reactive cysteine could be genetically introduced by mutation.^[3a]^ Maleimide is utilized in a reaction with cysteine to provide one of the most popular modifications in which fluorophores can be used to modify antibodies.^[3b]^ However, these bioconjugation methods can only be used for protein modification i*n vitro*. In order to achieve fluorescent labeling in the live‐cell, fluorescent proteins (FP), peptides^[4]^ or protein tags, and unnatural amino acid incorporation are conceivable (Figure 1b).

One of the most broadly researched and applied proteins in biochemistry and cell biology experiments is the Green fluorescent protein (GFP). GFP was discovered in 1962 by Shimomura and colleagues, which is extracted from the jellyfish *Aequorea victoria*.^[5]^ After that, Shimomura accurately predicted the chromophore of fluorescent protein.^[6]^ Since the discovery of GFP,^[1]^ manifold engineered versions of GFP have been developed, including enhanced GFP, cyan fluorescent protein, blue fluorescent protein, red fluorescent protein and yellow fluorescent protein for multicolor imaging.^[2]^ However, FP typically has broad absorption and emission spectra, which cannot allow simultaneous monitoring of too many different proteins when using multicolor imaging. In addition, its photostability and fluorescence intensity are not high enough to be applied in super resolution imaging (SRI) such as Stimulated Emission Depletion (STED) microscopy or single molecule imaging (Figure 1c). In contrast, organic dyes with narrow spectra, high fluorescence intensity and better photostability are more widely used. By means of chemical labeling, the protein of interest (POI) can append different chemical dyes. In live‐cell imaging techniques, microscopy, fluorophores, and protein tags are the main issues that need to be considered. This review concentrates on various protein labeling strategies and technologies in modern fluorescence imaging, for example, HaloTag, SNAP‐tag, TMP‐tag and unnatural amino acid systems and their applications. While previous reviews catalog the developments in this area,^[7]^ in this review, we not only provide timely updates on recent advances but also suggest a selection guide for users to choose their ideal labeling methods, directions, as well as instructions for designing novel protein tags. With the vast potential of chemical tag technology, we hope that protein labeling strategies with sophisticatedly designed fluorescent dyes can be the next generation tool for researchers to explore complex biological processes and tackle insurmountable challenges at the cutting edge of biological research.

## 2 HaloTag

**FIGURE 2:**
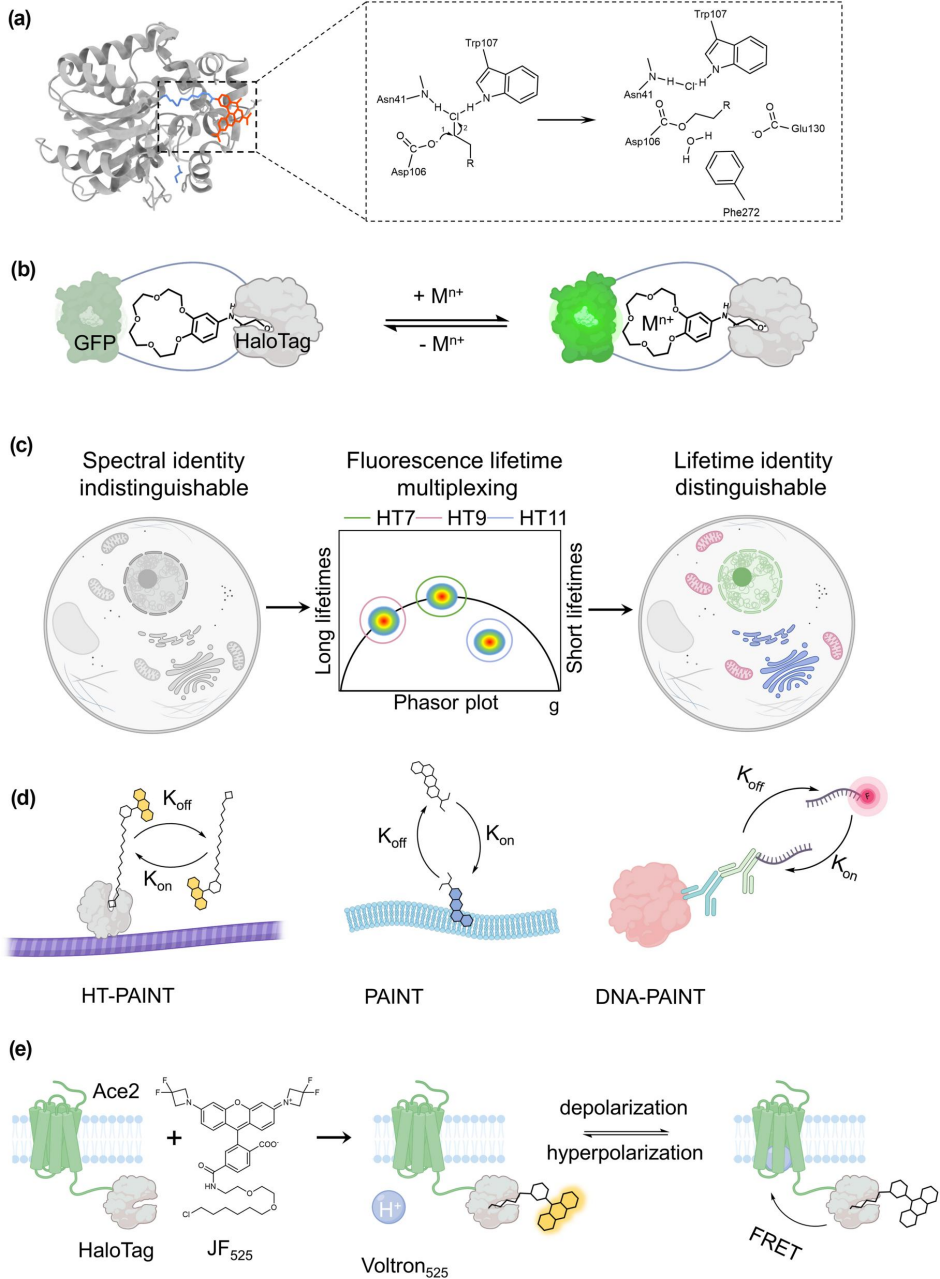
HaloTag system. (a) Labeling mechanism of HaloTag and the crystal structure of HaloTag7‐TMR (PDB ID: 6y7a). (b) Schematic figure illustrating the chemigenetic fluorescent Ca^2+^ indicators. (c) Schematic figure illustrating the fluorescence lifetime using one dye for multiple cellular organelles. (d) Schematic figure illustrating the HT‐PAINT, PAINT, and DNA‐PAINT. (e) Voltron, a fusion of Ace2 and HaloTag labeled with JF525 dye, can sense membrane potential. The scheme was partially created with BioRender.com.

HaloTag is a 33‐kDa self‐labeling tag that is revolutionized from a bacterial haloalkane dehalogenase (DhaA, *Rhodococcus*), developed by Promega in 2008.^[8]^ DhaA removes halides from hydrocarbons through bimolecular nucleophilic substitution in which the aspartate residue in the enzyme forms a covalent ester bond with the alkylogen substrate and then histidine mediates the hydrolysis of the intermediate to an alcohol before releasing it.^[9]^ By replacing His272 with catalytically inactive phenylalanine, the mutant DhaA irreversibly catches the ester intermediate in the previous step (Figure 2a).[^1^, ^8^] HaloTag is a powerful tool that can label fusion proteins with rapid labeling kinetics (conjugated with TMR, 1.88 × 10^7^ M^−1^s^−1^),^[10]^ high specificity (prokaryote origin, much less abundant in mammalian cells) and stability (ΔG_folding_ = −5.6 kcal/mol).^[11]^ However, compared to other self‐labeling tags, the large size would interfere with the function of the POI.^[12]^

Currently, several HaloTag ligands are offered for different applications ranging from biomolecular and biological‐process imaging^[13]^ to protein manipulation.^[14]^ By linking the fluorescent dyes like Atto655 to the HaloTag ligand, HaloTag technology can be applied to investigate protein localization^[15]^ and dynamics,^[16]^ to visualize cytoskeleton^[17]^ and tumors in various colors in live animals.^[18]^ Besides proteins, metal cations play essential roles in physiological events of cells and tissues. Tethering the fluorophore and different synthetic ion indicators to the HaloTag ligand is a maneuverable strategy to visualize cation dynamics with protein‐specificity inside live‐cells. Utilizing this method has allowed the creation of a wide variety of synthetic calcium indicators. For example, Red‐Halo2,^[19]^ RhoCa‐Halo,^[20]^ and the far‐red indicator NIR‐Halo2^[19]^ are recently developed tools. However, these probes' poor cell permeability and solubility as well as the need for additional washing processes to get rid of unlabeled probes severely restrict their usefulness. With the maturation of fluorophore chemistry, the Johnsson lab and Lavis lab have recently developed fluorogenic versions of localizable Ca^2+^ indicators based on MaP dyes and JF_646_ fluorophores that enable washing free Ca^2+^ imaging.^[21]^ It was recently discovered that the enormous and fast alteration in fluorescence intensity is produced as a result of the fusion of HaloTag with protein sensor domains which would go through conformational transform in the vicinity of the attached dye.^[22]^ The Schreiter and Lavis groups created chemigenetic fluorescent Ca^2+^ indicators that are based on the genetic insertion of circularly permuted (cp) GFP into HaloTag with a ligand containing the Ca^2+^ chelator (Figure 2b).^[23]^ In the case of Zn^2+^ detection, Fluorescein‐based Zn^2+^ indicator ZIMIR‐HaloTag,^[24]^ ZP1‐HaloTag^[25]^ and coumarin‐based Zn^2+^ indicator ZnDA‐2H, ZnDA‐3H^[26]^ are novel fluorescent probes for quantitative mapping of [Zn^2+^] in targeted subcellular structures, which displayed several folds fluorescence enhancement upon Zn^2+^ binding. Beyond fluorescence imaging, by substituting fluorescent dye into the hydrophobic group, transmembrane HaloTag fusion proteins induce the degradation of cytosolic through the cellular quality control mechanism.^[27]^ These indicators often “turn on” when sensing ions via a mechanism that utilizes photoinduced electron transfer (PeT), while other techniques that have been used for the development of ratio‐based indicators include internal charge transfer (ICT) or fluorescence resonance energy transfer (FRET). These designs hold the possibility of delivering a scalable answer to the problem of protein‐specific detection of cations or other compounds.

HaloTag has been employed for decades with few deficiencies in various fields. Lately, Johnsson and coworkers introduced HaloTag variants HaloTag9, HaloTag10, and HaloTag11 engineered from the rhodamine binding site of HaloTag7. When tagged with rhodamines, the brightness and fluorescence lifetime of these variations either increased or reduced compared with HaloTag7. This enabled users to regulate the fluorescence intensity and fluorescence lifetime of rhodamines when applied to fluorescence lifetime imaging microscopy (FLIM) (Figure 2c).^[28]^ Another fascinating refurbishment of the HaloTag system is the development of exchangeable HaloTag ligands (xHTL) and dHaloTag7, which were designed from HaloTag7 by mutating the active‐site residue Asp106 into Ala. Applications in MINFLUX, PAINT, as well as multi‐frame, live‐cell STED microscopy are just some of the examples of how the exchangeable ligands opened up brand‐new imaging options for commonly utilized labeling techniques. In addition, dual‐color imaging may be achieved in PAINT and STED microscopy by combining orthogonal pairs of xHTLs with the fluorescent labels HaloTag7 and dHaloTag7.^[29]^ As a functional extension of this approach, the Heilemann lab combined hydrophobic and protein−peptide interactions (PAINT and HT‐PAINT), xHTL and weak‐affinity DNA hybridization (DNA‐PAINT) to increase labeling flexibility. Using STED microscopy, they obtained high‐resolution images of six different structures in a cell with reduced photobleaching (Figure 2d).^[30]^ The multiplexing capabilities of fluorescence microscopy may be improved by switching from covalent labels to exchangeable labels and combining various labeling techniques. Apart from the live‐cell imaging applications discussed above, HaloTag is also used for in vivo imaging on small animal models such as Voltron (Figure 2e).^[31]^ Voltron is a novel voltage indicator that precisely tracks neuronal activity in living animals. The dye in Voltron binds to ACE2 (a voltage‐sensitive microbial rhodopsin domain)^[32]^ and HaloTag and changes in intensity when specific neurons are turned on, allowing the researchers to detect neural signals in the brain. Currently, the Voltron has been designed for mouse, *drosophila* and zebrafish. This technique can be applied to light‐plate microscopes and other kinds of optical microscopes, and scientists are developing variants of the Voltron to make it suitable for two‐photon imaging.

## 3 SNAP/CLIP TAG

**FIGURE 3:**
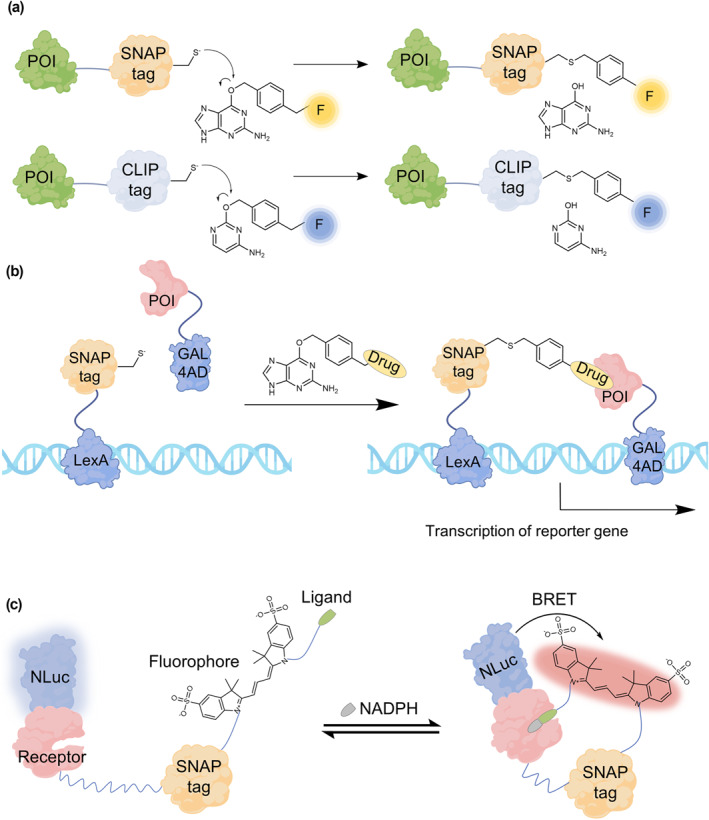
SNAP‐tag and CLIP‐tag systems. (a) Schematic diagram of SNAP‐tag and CLIP‐tag labeling mechanisms. (b) Schematic figure illustrating the SNAP‐tag–based Y3H system. (c) Design principle of a BRET sensor for detecting NADPH. In this system, a protein chimera between a SNAP‐tag, a receptor, and a luciferase (NLuc) is labeled with a ligand derivatized with a fluorophore. NADPH triggers ligand binding to the receptor, thereby increasing BRET. Abbreviations: F, fluorophore; NLuc, luciferase; POI, protein of interest; Y3H, yeast three‐hybrid. The scheme was partially created with BioRender.com.

SNAP‐tag is a 19.4 kDa self‐labeling tag, which was designed by Johnsson's lab in 2003. SNAP‐tag is engineered from human O^6^‐alkylguanine‐DNA alkyltransferase (hAGT).^[33]^ hAGT is a DNA repair protein that is capable of transferring the O6‐alkyl group from the alkylguanine substrate to the cysteine residue through the SN2 mechanism (Figure 3a).[^1^, ^34^] For improving the labeling efficiency and specificity for O^6^‐benzylguanine substrates (BG), Johnsson's group developed SNAP‐tag by employing random mutagenesis and direct evolution of hAGT.^[35]^ SNAP‐tag also accepts substrates in which the guanine is replaced by the more cell permeable O^4^‐benzyl‐2‐chloro‐6‐aminopyrimidine (CLP). Although CLP substrates show 4−14‐fold slower reaction kinetics than the corresponding BG substrates (Table 1),^[10]^ CLP conjugated with TMR incubation rapidly (5 min) generates fluorescence in living cells due to its preferable cell‐permeability.^[36]^ CLIP‐tag is a variant of the SNAP‐tag, which selectively reacts with the O^2^‐benzylcytosine (BC) derivatives instead of BG (Figure 3a).^[37]^ Due to the orthogonality of SNAP‐tag and CLIP‐tag, they are able to identify protein‐protein interactions in live‐cells by concurrently and precisely labeling with separate molecular probes. This allows for a more accurate analysis of the interaction between the two proteins.[^37^, ^38^] Although the labeling kinetics (BG‐TMR, 2.71 × 10^5^ M^−1^s^−1^)^[10]^ is not as fast as HaloTag, SNAP‐tag has less interference to the dynamics and native function of the POI due to the smaller size.

**TABLE 1:** Comparison between different protein tags.

Protein Tag	Size [kDa]	*t*_1/2_ (SiR, cell entry) [min]^a^	*k*_app_ (TMR) [M^−1^s^−1^]^a^	*K*_*d*_[M]^a^	N‐C distance [Å]^a^
Halotag	33	<3	(1.88 ± 0.01) × 10^7^	3.26 × 10^−7^	32.5
SNAP‐tag	19.2	BG:54	BG:(4.29 ± 0.01) × 10^5^	BG: 2.07 × 10^−6^	39.6
			CLP: (7.69 ± 0.01) × 10^4^	CLP: 1.669 × 10^−5^	
CLIP‐tag	21.8	‐	(0.81 ± 0.01) × 10^5^	1.67 × 10^−5^	‐
TMP‐tag3	18	9.7	1.1 × 10^6^	5.06 × 10^−10^	14.2

^a^ Data were referred to the literature[^10^, ^47^]

SNAP‐tag and CLIP‐tag are widely applied and effective methods for a broad range of applications. Based on their orthogonality, different molecular probes allow for the alternative and simultaneous labeling of these proteins in live‐cells.^[37]^ They can also be applied to detect the concentration change of a metabolite as a fluorescence resonance energy transfer (FRET)‐based biosensor.^[39]^ Not only did SNAP‐tag act as a protein tag in cell imaging,^[40]^ but also it can be applied to detect protein and drug interactions (Figure 3b).^[41]^ Johnsson's lab applied the SNAP‐tag to yeast three‐hybrid (Y3H) system for the profiling of FDA‐approved drugs. In this Y3H system, obscure drug‐protein interactions can be identified using affinity chromatography.^[41]^ Furthermore, SNAP‐tag can be engineered to semisynthetic sensor proteins utilized in paper‐based metabolic assays (Figure 3c). The metabolite is oxidized by nicotinamide adenine dinucleotide phosphate, and the presence of the reduced cofactor causes the sensor to change color, making it easier to measure the metabolite with a digital camera.^[42]^ This method permits the identification of several metabolites for quantitative point‐of‐care diagnostics, including phenylalanine, glucose, and glutamate. Combining various dyes into the SNAP‐tag ligands to design hybrid probes has become a commonly used strategy; for example, the micro‐viscosities of specific organelles can be detected by FLIM.^[43]^ Through direct stochastic optical reconstruction microscopy (dSTORM) imaging, this hybrid labeling approach that combines chemical probes with genetically encoded methods enables reconstructions of ER morphologies.^[36]^

## 4 TMP‐TAG AND OTHER TAGS

**FIGURE 4:**
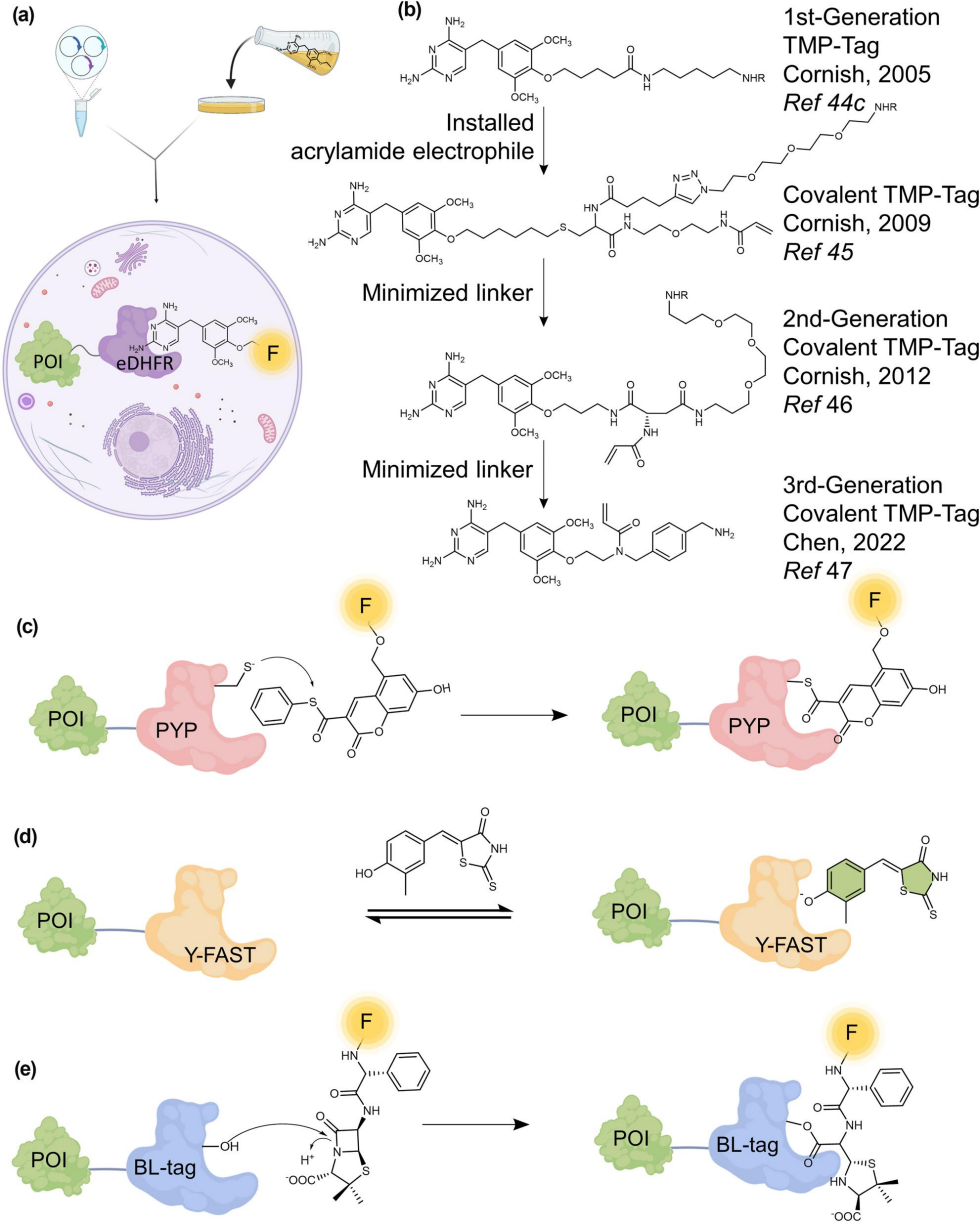
Additional chemical tags. (a) The TMP‐eDHFR system. (b) The chemical evolution of TMP‐Tag. (c)–(e) Labeling mechanism of PYP‐tag (c), Y‐FAST (d), and BL‐tag (e) fusion proteins. Abbreviations: F, fluorophore; POI, protein of interest. The scheme was partially created with BioRender.com.

TMP‐tag is a desirable technology among the self‐labeling tags that are now available. Beyond covalent interactions between enzyme and substrate, non‐covalent interactions between a drug and target protein have also been utilized in the design of protein tags due to the high affinity and selectivity of the interaction. The Cornish lab reported that the folate analog trimethoprim (TMP) can be used to alternatively label *E. coli* dihydrofolate reductase (eDHFR) fusion proteins in mammalian cell lines with low background (of mammalian DHFRs *K*_*d*_ > 1 μM) and fast kinetics (*K*_*d*_∼1 nM) (Figure 4a).^[44]^ Based on proximity‐induced reactivity, a covalent TMP‐tag was designed with a unique cysteine residue (eDHFR:L28C), which can react with an acrylamide electrophile.^[45]^ However, despite these technical improvements, the initial covalent TMP‐tag remained with several problems, such as slow in vitro labeling kinetics (*t*_1/2_ ∼ 50 min) and difficult to label proteins in live cells.[^45^, ^46^] After optimization, especially the Cys residue position and acrylamide trimethoprim‐fluorophore, the second‐generation covalent TMP‐tag had a rapid labeling half‐life (*t*_1/2_ = 8 min) and could easily label all manner of intracellular proteins in the living cell.^[46]^ TMP‐tag is being developed and used much more slowly than the concurrent SNAP‐tag and HaloTag. Until recently, our lab reported a third‐generation covalent TMP‐tag with significantly improved membrane permeability, fast labeling kinetics, and fluorogenic rhodamine dye compatibility.^[47]^ TMP, acrylamide, and the fluorophore are all components of this third‐generation tag with close distance.^[47]^ On the one hand, the linker between the dye and adapter is kept to a minimum because modern spironolactone/lactam rhodamine renders fluorogenicity by using residues on the surface of the protein.^[48]^ On the other hand, TMP was functionalized with a two‐carbon amine, and then immediately acrylate was attached to this nitrogen. Additionally, the PEG linker that was used in earlier designs was determined to be unnecessary and was consequently eliminated. The discovery of this kind of development not only improves the labeling technology that is already available but also makes it possible to see many living cells using more sophisticated microscopes (Figure 4b).^[47]^

Fluorogenicity is a commonly used property when designing a probe, because it enables a dye to “turn‐on” after labeling. Especially in complex biological environments, by reducing labeling background and enhancing the signal‐to‐noise ratio, a fluorogenic probe can be used. Currently, there are two primary strategies for fluorogenic dyes. The first approach involves the attachment of a quencher in close proximity to the fluorescent moiety. Upon binding to the target protein, the quencher dissociates, thereby inducing fluorescence in the probe. The second strategy entails the exploitation of an equilibrium between the open and closed form of a fluorophore, as exemplified by SiR dye^[48a]^ and elaborated by MaP^[49]^ and JF dye series.^[50]^ Thus, from the perspectives of biochemistry and cell biology, techniques for fluorogenic TMP‐tag are receiving a lot of interest.^[51]^ The first fluorogenic TMP‐tag, known as TMP‐Q‐Atto520, is a TMP‐fluorophore‐quencher molecule. The quencher is connected to a leaving group, and when TMP binds to eDHFR, it causes the leaving group to be cleaved by a cysteine residue that is placed just outside the binding pocket of eDHFR.^[52]^ By using an environmentally sensitive boron phenyldipyrromethene (BODIPY) dye, Liu et al. developed a rapid (*t*_1/2_–2 min) and fluorogenic labeling probe TMP‐AcBODIPY.^[53]^ Additionally, the Zhu group created a variety of TMP tag fluorogenic probes that cover the entire blue spectral region and have a quick reaction time (*t*_1/2_ = 33 s) and spectacular fluorescence increase.^[54]^ These probes, which included the ligand, the coumarin dye, and a quencher, effectively labeled proteins without the need for washing in both zebrafish and live cells. The modular structure of the fluorogenic TMP tag makes it easy to add multiple fluorophores with diverse characteristics and purposes.^[55]^ In addition to labeling proteins, TMP that has been conjugated with the organic fluorophore ATTO655 possesses high photon flux as well as rapid photoswitching capability in living cells. As a result, the dynamics of the H2B protein in live cells at a resolution of 20 nm may be easily observed using dSTORM.^[56]^ Except for dSTORM, TMP‐tag is also available for two‐photon imaging in living cells. This method now has access to a two‐photon fluorophore, which expands the repertoire of labels that may be used with it.^[57]^ Azido‐acyl caged oxazines have also been coupled with the TMP‐tag to label particular proteins in mammalian cells.^[58]^ Moreover, TMP‐tag has also been utilized in a variety of fluorescence‐related applications. By using a cell‐permeable photocaged dimerizer which was derived from TMP‐tag, Chenoweth and colleagues reported a novel method to reversible control of protein localization in living cells in a short period of time and subcellular spatial resolution can be achieved. This method broadens the scope of TMP‐tag application in the living cell.^[59]^

The PYP‐tag, developed by the Kikuchi group in 2009, is a 14 kDa self‐labeling tag employing the photoactive yellow protein (PYP) as the protein tag, which is naturally found in various purple bacteria such as *Halorhodospira halophila*.^[60]^ By trans‐thioesterification reaction with Cys69, PYP covalently binds to the derivatives of 4‐hydroxycinnamic acid and CoA, which is its natural substrate (Figure 4c).^[60]^ Recent creation of the Yellow Fluorescence‐Activating and Absorption‐Shifting Tag (Y‐FAST) was made possible by directed evolution of PYP. Y‐FAST is a 14‐kDa monomeric protein tag that was designed from PYP. It binds in a non‐covalent way highly permeable and nontoxic fluorogenic ligand hydroxybenzylidene rhodanine (HBR) derivatives (Figure 4d).^[61]^

Another tag developed by the Kikuchi group is the BL‐tag.^[62]^ TEM‐1 is a class A *ß*‐lactamase that hydrolyzes *ß*‐lactam antibiotics in an effective and specific manner. Examples of *ß*‐lactam antibiotics include penicillin, ampicillin, and cephalosporin. Eukaryotic cells do not have an indigenous equivalent for this enzyme. BL‐tag is a 29‐kDa protein tag that was generated from TEM‐1.^[63]^ After the acylation step of the reaction between TEM‐1 with *ß*‐lactams, ampicillin derivatives can covalently label to BL‐tag (Figure 4e).^[63]^ The Kikuchi group has broadened the range of applications for the BL‐tag technology by inventing a variety of fluorescent labeling probes. These probes allow for investigation into the activities of target proteins as well as live cell imaging.^[64]^ Further, utilizing FRET‐based fluorescein‐cephalosporin‐azopyridinium probes and a mutant BL‐tag, they were able to design a no‐wash fluorogenic labeling system.^[65]^ However, the acyl‐enzyme intermediate of the catalytically‐inactive TEM‐1 mutant (E166N) can be deacylated via intramolecular rearrangement leading to decreased labeling efficiency.^[66]^ Moreover, the substrates with labile *ß*‐lactam rings are chemically susceptible to acid, alkaline and other nucleophiles, so they must be handled carefully during synthesis and storage.^[67]^ In order to improve these shortcomings of BL‐tag, more recently, a new tag was created by the Kikuchi group. It is based upon a wild‐type TEM‐1 *ß*‐lactamase protein tag and fluorophore‐conjugated diazabicyclooctane *ß*‐lactamase inhibitors.^[68]^ The fluorescent probes were able to effectively create a stable carbamoylated complex with the *ß*‐lactamase, and the labeled proteins could be observed in living cells for an extended length of time.

## 5 UNNATURAL AMINO ACIDS SYSTEM

**FIGURE 5:**
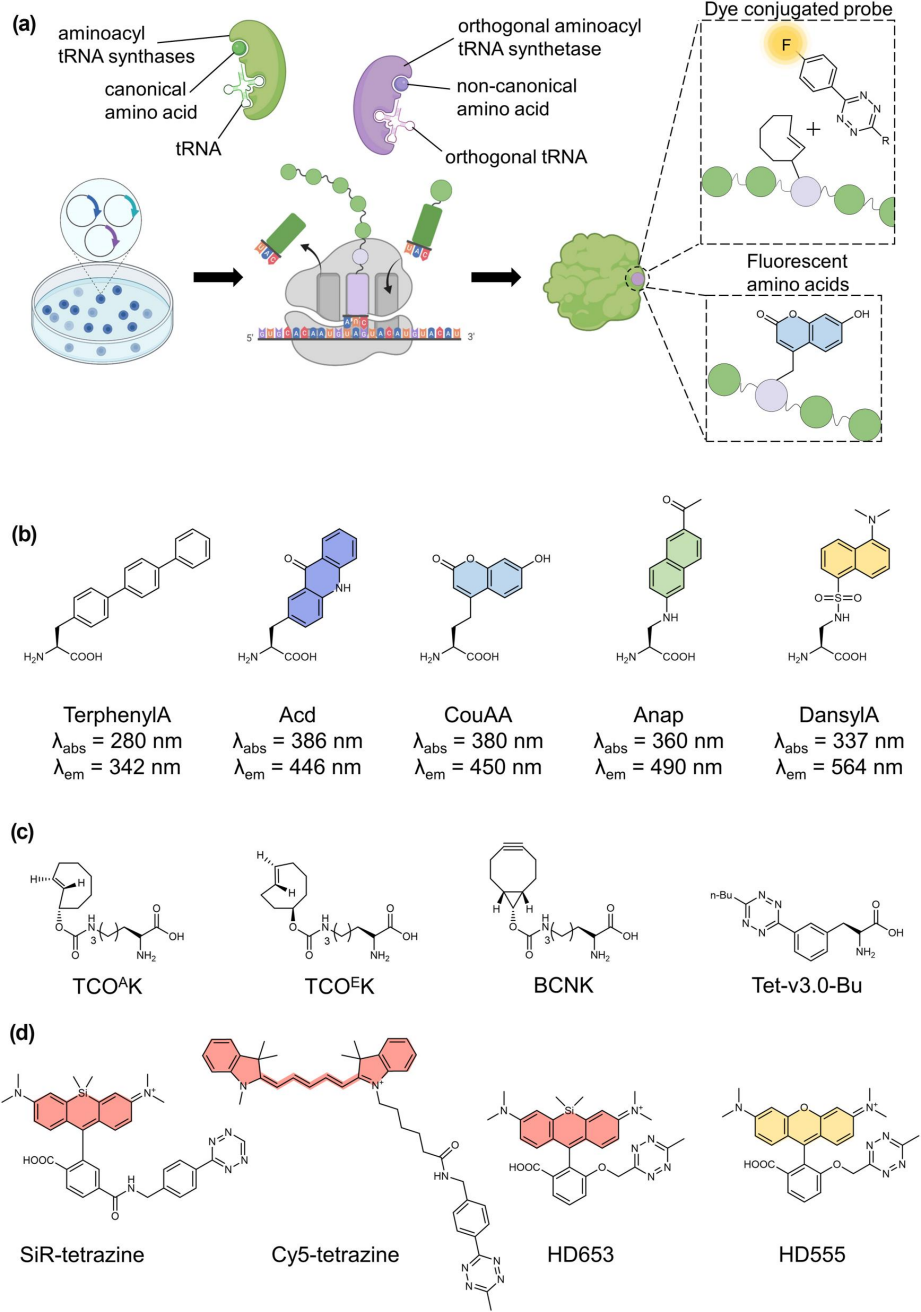
Labeling cellular proteins through GCE and click chemistry. (a) Schematic figure illustrating the ribosomal incorporation of a UAA into a protein. Protein labeling can be performed either through direct incorporation of fAAs or a two‐step strategy that the UAA with a clickable moiety can be site‐specifically conjugated with advanced fluorophores. (b) Properties of genetically encoded fAAs. (c) Chemical structures of selected UAAs that are compatible with click chemistry. (d) Structures of some clickable tetrazine dyes. Abbreviations: fAA, fluorescent amino acids; GEC, genetic code expansion; UAA, unnatural amino acid. The scheme was partially created with BioRender.com.

Almost all of these tagging techniques mentioned above may interfere with proteins or peptides. In order to obtain a more specific and clear biological structure, SRI requires a tag that is as small as possible and is able to couple to bright organic fluorophores that are photostable. This ensures the highest possible picture resolution. Among all the labeling strategies for fluorescence imaging, a technique that has just started to develop in recent years is the use of fluorescently labeled unnatural amino acids (UAAs), also known as genetic code expansion (GCE).^[69]^ Unlike the above‐mentioned labeling approach that requires the addition of protein/peptide bulk, this technique substitutes individual amino acids on the protein of interest by UAAs via an orthogonal aminoacyltRNA synthetase (aaRS)/tRNA pair (Figure 5a).^[70]^ GCE for protein labeling consists of UAAs, the aaRS/tRNA pair specific for the UAA, and genes with a nonsense codon at a designed site, which serve as the UAA incorporation position. Since there are 61 sense codons for only 20 natural amino acids, nonsense codons (for example, UAG, UGA and UAA, known as amber, opal and ochre, respectively) and quadruplet codons are chosen as encoding UAAs.^[71]^ The ever‐increasing level of sophistication of the technology has led to the development of a wide variety of orthogonal pairs of aaRSs and cognate tRNAs TyrRS/tRNATyr (from *Methanococcus jannaschii*) and an archeal PylRS/tRNAPyl pair from *Methanosarcina barkeri* or *Methanosarcina mazei* have been widely used and they are mutually orthogonal to each other.^[72]^

Protein labeling can be performed either through fluorescent amino acids (fAAs), which were directly incorporated into POI, or applying the UAA bearing a clickable moiety can be site‐specifically conjugated alternative fluorophores.^[73]^ In the direct labeling strategy, proteins can be incorporated in fAAs such as a coumarin‐derived amino acid (CouAA),^[74]^ acridonylalanine (ACD),^[75]^ (acetylnaphthalenyl) amino propanoic acid (Anap),^[76]^ dansylalanine^[77]^ and TerphenylA^[78]^ (Figure 5b). AnapRS/tRNA is necessary for the mammalian system to be able to encode the Anap protein, which undergoes a significant shift in emission maxima and fluorescence intensity depending on the circumstances of its surrounding environment.^[79]^ Such properties can be used to investigate protein dynamics and its subcellular localization.^[80]^ CouAA is another handy fAA that is environmentally sensitive with an altered pH in the solvent the absorption spectrum changes.^[74]^ Since the click chemistry was discovered, UAAs with a clickable functional moiety have enabled a more versatile two‐step labeling strategy. Given that strain‐promoted inverse electron demand Diels‐Alder cycloaddition (SPIEDAC) is regarded to be the commonly used bioorthogonal reaction with the quickest rate, it is widely utilized in fluorescent imaging.^[81]^ ncAAs for SPIEDAC include axial *trans*‐cyclooct‐2‐ene lysine (TCO^A^K),^[82]^ equatorial *trans*‐cyclooct‐4‐ene lysine (TCO^E^K),^[82c]^ *endo* bicyclo[6.1.0]nonyne lysine (BCNK),[^82^, ^83^] tetrazine‐based UAAs, such as Tet‐v3.0^[84]^ (Figure 5c). Of note, although TCO^A^K tends to be faster when reacting with tetrazines (∼10‐fold), BCNK has higher stability in cells.^[85]^ The interchromophore distance, an important factor in determining the intensity of a dye, between the tetrazine and the fluorophore determines quenching efficiency of tetrazine dyes, and quenching efficiency of far‐red‐shifted fluorophore is much lower than their blue‐shifted analogs.^[86]^ Therefore, SiR‐tetrazine,^[87]^ Cy5‐tetrazine,^[82b]^ HD653 and HD555^[88]^ are commonly used tetrazine dyes (Figure 5d).

The use of GCE labeling in site‐specific fluorescent placement to clarify the cellular locations and activities of proteins has been reported in recent investigations. Using confocal and dSTORM, the Choquet and Sauer groups used the GCE labeling strategy to visualize the different localization of the γ2 and the γ8, two AMPA receptor subunits. This technique enables fluorescently labeling protein epitopes which cannot be recognized by antibodies in live neurons and brain slices.^[89]^ Slavoff and coworkers incorporated BCNK bearing with SiR into alt‐RPL36 and discovered that alt‐RPL36 is increasing of PI3K‐AKT‐mTOR signaling.^[90]^ In other studies, Hang and colleagues utilized TCO^A^K and BODIPY to label IFITM3, revealing that IFITM3 enhances the transport of this pathogenic cargo to lysosomes upon fusion with incoming viral particles as well as the antiviral activity that occurs downstream, which is regulated by IFITM3 S‐palmitoylation.^[91]^ The application of fluorogenic probes can assuredly enhance signal‐to‐noise ratio and simplify bio‐imaging protocol by omitting additional washing steps. For a wide range of live‐cell fluorescent imaging and protein labeling applications, the Wombacher and Herten groups created a number of cell‐permeable and ‐impermeable tetrazine‐dye conjugates to target UAAs and facilitate no‐wash multicolor labeling of extracellular and intracellular region.^[88]^

## 6 HOW TO DESIGN A NEW TAG

In this review, we focus on tags that can covalently label intracellular proteins and are widely used in biological research. Designing a protein tag is generally inspired by the following patterns: receptor‐ligand, enzyme‐substrate, and enzyme‐inhibitor. In general, covalent labeling can be achieved by binding to a suicide inhibitor or by recognizing and transferring the substrate to a peptide motif that is appended to a protein.^[33b]^ Reminiscent to the covalent drug technology, a non‐covalent tag can be engineered to a covalent version through directed evolution^[8]^ or proximity‐induced reactivity.^[45]^ In theory, the vast chemical space of pharmaceutical chemistry can turn into more protein tags. However, a desirable tag for protein labeling in live cells needs additional considerations. Protein tags with small size are necessary. Monomeric, and stable may give minimal interference with the biological process under study. Under physiological conditions, it is important that the labeling process that takes place between the protein tag and the fluorescent ligand happens quickly (*k*_cat_/*K*_*M*_ > 10^6^)^[2]^ and nearly quantitative, and *K*_*M*_ is small enough to allow labeling of low concentrations of the POI. To achieve specificity, the tag should be exogenous and should not react with endogenous molecules. Under this condition, plant or bacterial peptides or proteins might be a good choice.^[7c]^ Finally, the ligands/substrates should have design flexibility that are compatible with cell‐impermeable and cell‐permeable fluorescent probes. Especially for the cellular protein targets, probe bioavailability is often the key to successful labeling. We regard HaloTag as the best example of cell permeability whose ligand can freely pass blood‐brain barrier.^[92]^ These considerations underscore the challenges of developing successful new tags.

On the protein side, protein engineering is progressively focusing on methods other than fusing tags at the N‐terminal or C‐terminal of POI, such as protein loop.^[93]^ This approach can be applied to proteins whose termini are not on the surface or proteins with specific functions at the N/C terminal. However, the architectural integrity of the POI and tag may be impacted by loop fusion. Therefore, the labeling strategy should be custom‐designed based upon the structure of the POI. Meanwhile, the N‐C distance of the tag protein needs to be short. TMP‐tag is a good example; the N‐C distance of HaloTag and SNAP‐tag is 32.5 Å and 39.6 Å, respectively, compared to eDHFR's 14.2 Å.^[47]^ Although the N‐C distance of HaloTag is long, it can be circularly permuted (cp) by introducing new termini near the fluorophore binding site at position 143.^[22]^ Conformational changes adjoining the attached fluorescent dye would result in substantial and fast variations in the fluorescence intensity when cpHaloTag was fused with protein sensor domains.^[22]^ Circular‐permutation technology is a promising strategy to generally devise protein sensors with tags, which would open a huge number of applications.

## 7 CONCLUSION AND FUTURE PERSPECTIVES

In this review, we have discussed emerging bioconjugation methods for modern fluorescence imaging with a particular focus on live‐cell labeling strategies and fluorescent probes. We highlighted the recent advancements in labeling techniques, probe design, and optical instruments that have boosted fluorescence imaging technologies toward higher spatiotemporal resolution and sensitivity. In parallel with the evolution of organic fluorophores that are brighter and more photostable, the development of novel labeling techniques such as the HaloTag, SNAP‐tag, TMP‐tag, and UAA system have allowed for precise and specific labeling of proteins within living cells, enabling researchers to monitor their behavior and study their functions in unprecedented detail. Labeling protein technology has become an essential part of the molecular biology arsenal as a result of its novel combination of features. Moreover, applications of tagged proteins are not limited to confocal microscopy and SRI but can also be applied to two‐photon fluorescence lifetime imaging. It is fair to say now that chemical tags have gradually become the go‐to option of advanced optical microscopy.

The era of fluorescent protein began in the 1990s has profoundly changed the way to perform biological research. Although the chemistry community has introduced various protein tags in recent years, their usage is far behind that of fluorescent proteins, which are simpler, more available, and cleaner. To realize the potential of introducing a brighter and enlarged dye palette to proteins using chemical tags, several challenges need to be addressed. First of all, the labeling efficiency of chemical tags hinges on the permeability/bioavailability. Only if a near‐quantitative labeling can be achieved in live cells or even animals could the tags be superior to the fluorescent protein counterparts, and this is a formidable goal. Second, the specificity of the probe and the compatibility of the bulky protein tag are yet to be fully established. The last issue is non‐scientific, but the efficient distribution of chemical reagents is extremely cumbersome. Unlike plasmids that can be amplified and distributed through a third‐party platform like Addgene, dyes have to be manually synthesized repeatedly to try to adapt. The openness of the tag community, in conjunction with successful commercialization, would be the key factor to further popularize chemical tags.

An ultimate goal of the field of tags is to have the tag sequence as minimal as possible. The FlAsH/ReAsH^[4]^ tags and PRIME^[94]^ labeling methods are significant advances along this line, but the cost is less specific labeling or additional enzymes. The Amber suppression strategy allows the incorporation of UAAs into a POI. The resilience and adaptability of this method pave the way for a variety of possibilities that will be intriguing to investigate, among other things, multicolor imaging of the nanoscale structure, interactions, and transport of intracellular and/or extracellular proteins in living cells. This will be one of the areas of investigation that will be of particular interest. Stoichiometric labeling can be achieved even at sterically protected protein locations thanks to the little disturbance of POI that is caused by the incorporation of a single UAA. This is made possible by the tiny size of tetrazine‐based dyes. Due to the fact that it has a sterically low need for the labeling, the approach is especially useful for quantitative super‐resolution microscopy. However, there are still certain barriers to overcome in terms of the capability to show a saturation of the labeling.^[89]^ The main difficulty with this method has been how inefficiently the full‐length protein bearing the UAA can be produced.^[95]^ Even though multicolor GCE may be applied to two color labeling, doing so is very challenging since using two separate ncAAs calls for not only two orthogonal click‐reactions but also two mutually orthogonal tRNA/RS combinations that can specifically integrate two different clickable ncAAs. Nonetheless, we are looking forward to the advances along these lines, which would finally bring the labeling accuracy beyond nanometer precision.

Finally, we offer a selection guide for users to choose their ideal labeling methods. Among all these tags, HaloTag is one of the earliest commercialized protein tags. During the live cell staining, cell membrane permeability may become the decisive step when working with intracellular targets. As shown in Table 1, the HaloTag ligand offers the fastest staining speed and high signal‐to‐noise ratio among other tags, making it a first choice for labeling proteins in live cells. These advantages have contributed to the widespread application of HaloTag in recent years, not only for protein and metal ion imaging but also in other biophysical applications.^[96]^ However, in some cases, the large size of HaloTag may interfere with the expression of the POI, so relatively small protein tags like TMP‐tag and SNAP‐tag can be considered as alternatives. In comparison to HaloTag, the *in cellulo* labeling rates of the BG ligand of the SNAP‐tag are much slower. Due to hydrogen bonding effects of BG ligand, it has strong aggregation tendency in aqueous media and very low water solubility,^[36]^ leading to its lowest permeability when conjugated to SiR dye (Table 1). On the other hand, despite 4−14‐fold slower reaction kinetics in vitro, the CLP ligand has more rapid and robust labeling of SNAP‐tag than the corresponding BG ligand in living cells.^[97]^ TMP‐tag3 labels cellular targets more effectively than SNAP‐tag and has better cell permeability. With the shortest N‐C distance among these three tags, the DHFR in TMP‐tag3 is a well‐studied model protein for loop fusion bioengineering. However, the current TMP‐tag3 still requires the addition of a NADPH, complicating the protocol. Depending on the biological questions of interest, researchers need to choose an appropriate protein tag to label POI with small molecule probes, which enables them to watch and alter protein activity. It is noteworthy that the HaloTag, SNAP‐tag, and TMP‐tag are mutually orthogonal; therefore, they can be exploited together in multi‐color labeling and imaging. In the future, with the increasing demand for imaging precision, especially with the development of MINFLUX nanoscopy, the large size of protein may eventually affect the accurate localization of the target protein or interfere with the function of the POI. By then, despite bearing the most complicated protocol among all the discussed methods, UAA would become a viable option in the future era of nanoscopy.

## CONFLICT OF INTEREST STATEMENT

Zhixing Chen is an inventor of the third‐generation TMP tag technology (patent pending).

## Data Availability

Data are openly available in a public repository.
